# Unique Dental and Craniofacial Manifestations of Hypoplastic Amelogenesis Imperfecta in a Patient With Prune Belly Syndrome: A Rare Case Report

**DOI:** 10.1155/crid/1724807

**Published:** 2025-09-25

**Authors:** Jan Ching Chun Hu, Jung-Wook Kim

**Affiliations:** ^1^Department of Biologic and Materials Sciences & Prosthodontics, University of Michigan School of Dentistry, Ann Arbor, Michigan, USA; ^2^Department of Pediatric Dentistry, School of Dentistry and Dental Research Institute, Seoul National University, Seoul, Republic of Korea; ^3^Department of Molecular Genetics, School of Dentistry and Dental Research Institute, Seoul National University, Seoul, Republic of Korea

**Keywords:** aberrant hypoplastic amelogenesis imperfecta, craniofacial dysmorphism, prune belly syndrome, whole exome sequencing

## Abstract

**Introduction:** Prune belly syndrome (PBS) is a rare congenital disease characterized by hypoplastic abdominal wall muscles, urological abnormalities, and bilateral cryptorchidism. This report describes a rare case of aberrant hypoplastic amelogenesis imperfecta in a patient with PBS.

**Case Presentation:** A 10-year-old boy with PBS presented with difficulties in speech and mastication. Oral and radiological examinations revealed aberrant hypoplastic enamel defects, mandibular hypoplasia and retrusion, maxillary constriction, and anterior open bite.

**Conclusion:** Although dental manifestations are not frequent in PBS, rare cases may present with aberrant hypoplastic amelogenesis imperfecta.

## 1. Introduction

Prune belly syndrome (PBS) is a rare congenital disorder primarily affecting males, characterized by the classic triad of features: wrinkled, prune-like ventral abdominal skin with underlying flaccid hypoplastic skeletal muscle, urinary tract dilation due to poorly formed contractile smooth muscle, and bilateral intra-abdominal cryptorchidism [1]. The condition is also known as Eagle–Barrett syndrome or triad syndrome, with a prevalence of approximately 1 in 25,000 or 3.8 per 100,000 individuals [2].

The genetic etiology of PBS remains heterogeneous, likely due to clinical and genetic locus heterogeneity. Several genes, including *CHRM3*, *STIM1*, *FLNA*, and most recently, *PIEZO1*, have been suggested as candidate genes [1, 3]. Additionally, some pathogenic copy number variants (CNVs) have been proposed as contributing genetic factors [2]. These associations require further validation.

Dental manifestations in PBS are neither consistent nor frequent findings; however, several reports have described conditions such as enamel hypoplasia and congenitally missing teeth, horizontal linear enamel hypoplasia and generalized hypocalcification with yellowish discoloration, and cleft lip [4, 5]. In this report, we present a rare case of aberrant hypoplastic amelogenesis imperfecta in a patient with PBS.

## 2. Case Report

**Figure 1:**
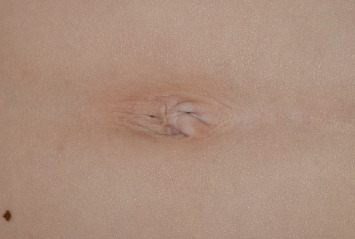
Wrinkled ventral abdominal skin visible around the belly button.

A 10-year-old boy presented to the Department of Pediatric Dentistry at Seoul National University Dental Hospital with chief complaints of difficulty in speech and mastication. The patient had a known diagnosis of PBS and exhibited wrinkled ventral abdominal skin (Figure 1), mild pectus excavatum, and right-sided esotropia. His medical history included bilateral cryptorchidism and hypospadias, both treated surgically. He also had persistent proteinuria, which was managed medically.

**Figure 2:**
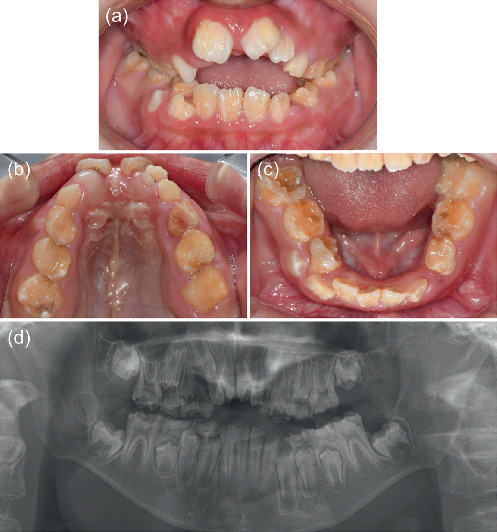
(a–c) Intraoral photographs of the patient at 10 years and 3 months of age. (d) Panoramic radiograph at 10 years and 3 months of age.

**Figure 3:**
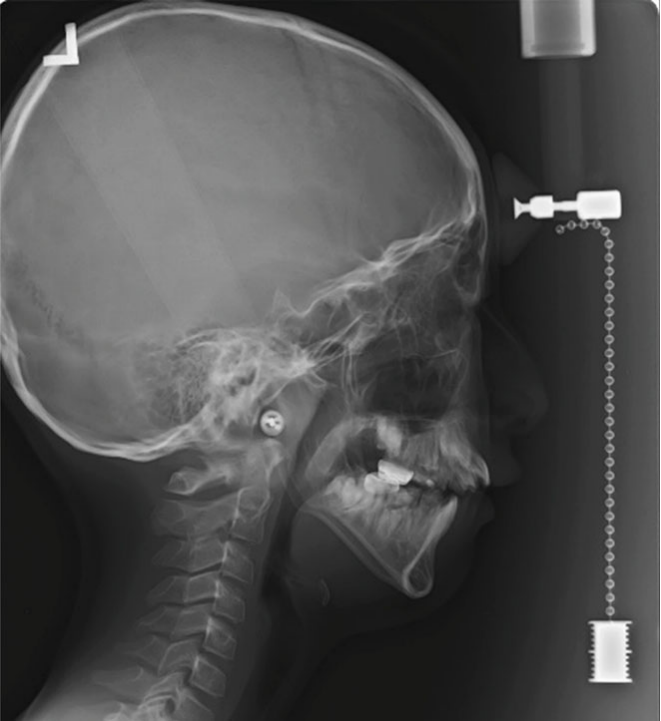
Cephalometric radiograph at 10 years and 11 months of age.

**Figure 4:**
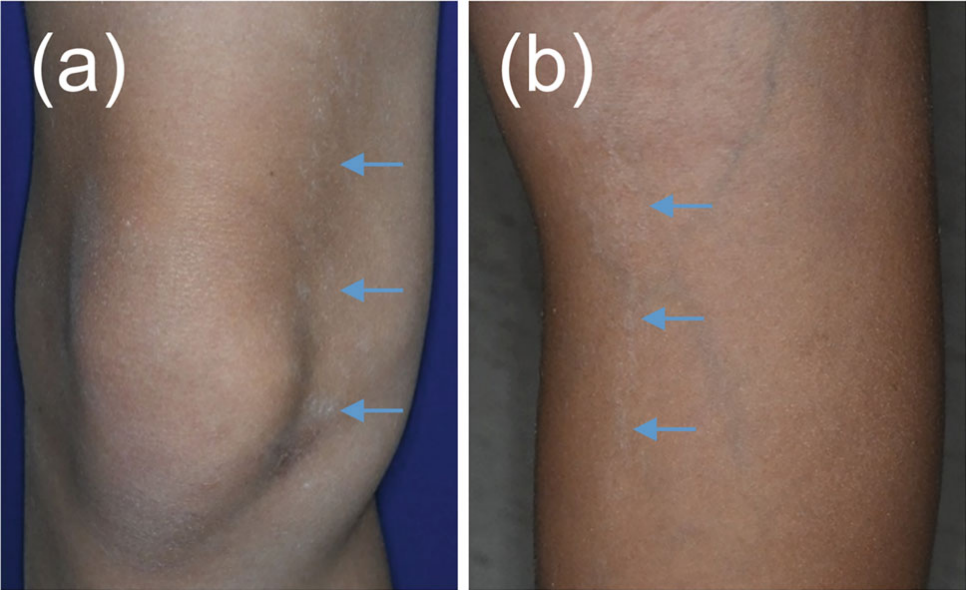
(a) Irregular white circular skin lesions (blue arrows) on the anterior medial aspect of the right knee. (b) Long vertical linear lesions (blue arrows) on the posterior medial aspect of the right knee.

Aberrant hypoplastic enamel defects were evident in almost all teeth, including developing dentitions (Figures 2, 2, 2, and 2). The mandible was hypoplastic and retrusive, and the maxilla was constricted with an anterior open bite (Figure 3). Additionally, irregular circular skin lesions on the anterior surface of the knees and vertical, linear lesions on the posterior surface of the knees were observed (Figure 4).

Genetic analysis was conducted using whole-exome sequencing data from the trio (comprising the healthy father and mother), as previously described [6]. Briefly, paired-end sequencing reads from the trio were trimmed and aligned to the human Genome Assembly GRCh38. Annotated variant lists were generated following the application of bioinformatic tools such as SAMtools, GATK, and ANNOVAR. Variants with a minor allele frequency greater than 0.01 were excluded. The analysis did not reveal pathogenic variants in previously suggested candidate genes for PBS. No candidate variants were identified through de novo or sex-linked inheritance analyses. However, two candidate variants in the *PLEC* and *OBSL1* genes were identified during autosomal recessive inheritance analysis (Table 1).

## 3. Discussion

This case highlights the co-occurrence of irregular hypoplastic amelogenesis imperfecta with PBS, expanding the documented phenotypic spectrum of PBS-related ectodermal anomalies. While dental manifestations in PBS remain rare, the findings of our case align with prior observations of enamel hypoplasia and structural dental defects in PBS patients [4, 5]. Although the enamel hypoplasia is irregular and aberrant, all teeth examined clinically or radiographically were affected. From the WES dataset, none of the candidate genes for amelogenesis imperfecta showed damaging variants. The identification of candidate variants in *PLEC* (involved in cytoskeletal integrity and skin/muscle development) and *OBSL1* (linked to skeletal growth and craniofacial patterning) offers additional insights into potential genetic contributors to PBS-associated ectodermal dysplasias [7, 8]. Functional studies are warranted to validate these associations and define the potential roles of *PLEC* and *OBSL1* in enamel formation and PBS pathogenesis. This case underscores the importance of comprehensive dental evaluations in patients with rare disorders to address functional impairments and improve quality of life through interdisciplinary care.

## Figures and Tables

**Table 1:** Candidate variants from whole-exome sequencing analysis.

**Gene (transcript) (location)**	**Variant**	**dbSNP**	**Allele**
*PLEC* (NM_201378) (8q24.3)	c.13453G > T, p.A4485S	rs960573896	Paternal
	c.4270C > T, p.R1424W	rs531928668	Maternal
*OBSL1* (NM_01531) (2q35)	c.3850G > A, p.G1284S	rs74589174	Maternal
	c.3395delA, p.E1132Gfs⁣^∗^7	Novel	Paternal

## Data Availability

The data that support the findings of this study are available on request from the corresponding author. The data are not publicly available due to privacy or ethical restrictions.
